# Beyond Respiratory Support: Clinical Profiles and Factors Associated with Mortality in Heart Failure Patients Admitted to the ICU

**DOI:** 10.3390/jcm15041334

**Published:** 2026-02-08

**Authors:** Duygu Kayar Calili, Suleyman Kalayci, Iffet Tiftikci, Elif Unal Kaya, Demet Bolukbasi, Seval Izdes

**Affiliations:** 1 Department of Anesthesiology and Reanimation-Intensive Care, Faculty of Medicine, Ankara Yıldırım Beyazıt University, Ankara Bilkent City Hospital, Ankara 06800, Turkey; sevalizdes@aybu.edu.tr; 2 Department of Cardiology, Ankara Bilkent City Hospital, Ankara 06800, Turkey; dr_suleyman_kalayci@yahoo.com; 3 Department of Intensive Care, Ankara Bilkent City Hospital, Ankara 06800, Turkey; iffettiftikci@gmail.com (I.T.); mdelifunal@gmail.com (E.U.K.); de.emeet@hotmail.com (D.B.)

**Keywords:** critically ill, heart failure, mortality, respiratory therapy, oxygen, mechanical ventilation, noninvasive, invasive

## Abstract

**Background:** Patients with heart failure (HF) admitted to the intensive care unit (ICU) often require respiratory support, yet comparative data on different respiratory modalities in this population remain limited. The aim of our study was to analyze clinical characteristics and outcomes of HF patients in the ICU receiving various respiratory therapies, while also identifying factors associated with mortality. **Methods:** This retrospective observational study categorized patients (*n* = 692) based on their respiratory treatments into four groups: no treatment (T-NR; *n* = 280); oxygen therapy (T-O_2_; *n* = 220); non-invasive mechanical ventilation (T-NIMV; *n* = 99); and invasive mechanical ventilation (T-IMV; *n* = 93). The groups were compared in terms of clinical characteristics, laboratory values at ICU admission and discharge, length of stay, and outcomes. **Results:** The T-IMV group had significantly higher Acute Physiology and Chronic Health Evaluation II (APACHE II) and updated Charlson Comorbidity Index scores, alongside lower ejection fractions and higher rates of vasopressor/inotrope use (*p* < 0.05). This group also exhibited higher mortality, longer ICU stays, and more adverse laboratory profiles (elevated lactate, creatinine, N-terminal pro-B-type natriuretic peptide-NT-proBNP, bilirubin; lower albumin). Multivariable analysis confirmed that the APACHE II score, vasopressor/inotrope requirement, and lactate and NT-proBNP levels (at admission) were independently associated with mortality (*p* < 0.05). **Conclusions:** Patients requiring IMV demonstrated greater severity of illness, as reflected by higher APACHE II scores, elevated NT-proBNP and lactate levels at admission, as well s increased requirement for vasopressor/inotrope therapy. These variables were associated with mortality, suggesting that the poorer prognosis observed in the IMV group was due to their underlying disease burden.

## 1. Introduction

Heart failure (HF), particularly in the setting of acute decompensation or concomitant respiratory failure, is associated with high comorbidity and mortality [1]. Managing critically ill HF patients in the intensive care unit (ICU) is complex and often involves respiratory therapies and multiple organ support systems [2,3]. In decompensated patients, life-threatening pulmonary edema can develop rapidly, progressing to severe respiratory failure and necessitating intensive cardiopulmonary support. While invasive mechanical ventilation (IMV) may be required in severe cases, the role of noninvasive approaches (such as conventional oxygen therapy and noninvasive mechanical ventilation (NIMV)) in reducing the work of breathing and managing cardiogenic pulmonary edema continues to be debated in clinical practice [2,4].

Although previous studies have evaluated respiratory therapies used in HF patients from various perspectives, a comprehensive comparative analysis of respiratory support methods in a homogeneous cohort of critically ill HF patients admitted to the ICU has not yet been performed [1,5,6,7]. Despite advances in treatment strategies for critically ill HF patients, high mortality rates persist. Therefore, identifying factors independently associated with mortality is crucial to improving risk stratification, guiding clinical decision making and ultimately increasing survival rates [8,9,10,11]. The current literature often examines these treatments or outcomes in isolation or within broader critically ill populations, leaving a gap in the context of a dedicated HF ICU cohort. Therefore, this study was designed with two primary objectives. First, we aimed to compare the clinical characteristics, treatments, laboratory parameters, ICU and hospital lengths of stay (LOS), and outcomes of patients admitted to the ICU with a diagnosis of HF, stratified according to the modality of respiratory support received. Second, we sought to identify independent factors associated with mortality among all HF patients in this cohort.

## 2. Materials and Methods

### 2.1. Patients

This retrospective observational study reviewed the data of adult patients admitted to coronary ICU with a diagnosis of HF between October 2020 and October 2022. Patients were admitted to the CICU based on the clinical diagnosis of acute decompensated HF requiring specialized care, such as intensive hemodynamic monitoring, management of severe hemodynamic instability, or respiratory failure. The study was conducted in accordance with the Declaration of Helsinki and was approved by the Ankara Bilkent City Hospital Ethics Committee (No: E2-22-2870). Exclusion criteria were defined as follows: (1) presence of pathologies causing acute respiratory failure other than HF decompensation, such as pneumonia or acute exacerbations of obstructive/restrictive lung diseases; (2) prior use of home respiratory therapy (oxygen therapy or NIMV); (3) ICU length of stay less than 24 h; and (4) readmissions within the same treatment period. Acute exacerbations or alternative causes of acute respiratory failure were identified and excluded based on detailed review of medical records, ICD codes, treatment protocols, and radiological imaging. The patients included in the study were categorized into four groups based on the respiratory treatment modalities applied: those receiving no respiratory support (treatment: non-respiratory, T-NR), those receiving oxygen therapy (treatment-oxygen, T-O_2_), those treated with NIMV (treatment: NIMV, T-NIMV), and those requiring IMV (treatment: IMV, T-IMV). Oxygen therapy was administered according to routine clinical practice and physician discretion. The T-O_2_ group included patients receiving conventional oxygen supplementation via nasal cannula, face mask, or high-flow nasal cannula. Patients in the T-NR group were admitted to the ICU for close hemodynamic monitoring and management of acute decompensation, including severe volume overload, hypotension, arrhythmias, or the need for vasopressor/inotropic therapy, despite not requiring ventilatory support at the time of admission.

### 2.2. Data Collection

The groups were compared in terms of age, sex, Acute Physiology and Chronic Health Evaluation (APACHE) II scores, and the updated Charlson Comorbidity Index (uCCI). Comparisons were also made regarding ICU and hospital length of stay (LOS; days), comorbidities (hypertension (HT), diabetes mellitus (DM), chronic kidney disease (CKD), pulmonary disease (PD), and cerebrovascular disease (CVD)), HF etiology (ischemic or non-ischemic), echocardiographic parameters [pulmonary artery pressure (PAP mmHg) and ejection fraction (EF%)), the administration of vasopressor/inotropic therapy, and invasive cardiological interventions (intra-aortic balloon pump (IABP), implantable cardioverter-defibrillator (ICD), extracorporeal membrane oxygenation (ECMO), and cardiac resynchronization therapy (CRT)). PD was defined as a documented medical history of chronic obstructive pulmonary disease, asthma, interstitial lung disease, or bronchiectasis, based on physician diagnoses and ICD-10 codes recorded in the patient’s electronic health records. Echocardiographic parameters were obtained through standard transthoracic echocardiography (TTE) performed within the first 24 h of ICU admission by experienced cardiologists. EF was measured using the modified Simpson’s method, while PAP was estimated based on the tricuspid regurgitation peak velocity using the Bernoulli equation. Serum levels of N-terminal pro-B-type natriuretic peptide (NT-proBNP pg/mL), lactate (LAC mmol/L), creatinine (Cre mg/dL), albumin (Alb g/L), and total bilirubin (TBIL mg/dL) were also recorded at two time points: ICU admission (T0) and ICU discharge or the last measurement before death (T1). These laboratory values were also compared among the groups. For the secondary objective of the study, the clinical characteristics and laboratory parameters of survivors and non-survivors were compared.

### 2.3. Statistical Analysis

Statistical analyses were conducted using IBM SPSS Statistics version 27.0 (IBM Corp., Armonk, NY, USA) and MedCalc version 15.8 (MedCalc Software bvba, Ostend, Belgium). Numerical data are expressed as the mean ± standard deviation or the median (interquartile range [IQR]), while categorical data are presented as frequencies and percentages. When comparing two independent groups of numerical data, the Mann–Whitney U test was conducted, while for categorical data, Pearson’s chi-square and Fisher’s exact tests were conducted. For comparisons involving three or more groups in the chi-squared test, cellwise residual analysis was applied as a post hoc method. To assess significance between the means of three or more groups, the Kruskal–Wallis test was performed for non-normally distributed numerical data, and one-way ANOVA for normally distributed parameters. Normality analysis was performed using the Kolmogorov–Smirnov test. To identify factors associated with mortality, univariable logistic regression analyses were first conducted, followed by multivariable logistic regression analyses, including variables that were clinically relevant and/or statistically significant. CKD (due to the risk of collinearity with admission creatinine levels) and LOS (as it represents a post-admission outcome rather than a baseline variable associated with mortality) were not included in the logistic regression model. Due to the near-perfect prediction of mortality by respiratory treatment modality (quasi-complete separation), this variable was omitted from the multivariable model. The decision was further justified by the presence of strong confounding by indication. The primary analysis was performed using variables obtained exclusively at admission (T0). This approach was adopted to prevent outcome leakage and to develop a clinically applicable model. The NT-proBNP variable was included in the model on a logarithmic scale to account for its right-skewed distribution and to more accurately reflect its proportional association with mortality. We present the absolute NT-proBNP values in descriptive tables and group comparisons to maintain clinical interpretability. Model discrimination was assessed using the area under the receiver operating characteristic curve (AUC), while calibration was evaluated using the Hosmer–Lemeshow test and calibration plots. Model explanatory power was reported using the Nagelkerke R^2^. Internal validation of the primary model was performed using bootstrap resampling with 1000 iterations. Variance inflation factor (VIF) analysis was performed to assess multicollinearity among variables included in the T0 multivariable regression model. A *p*-value of less than 0.05 was considered statistically significant

## 3. Results

**Figure 1:**
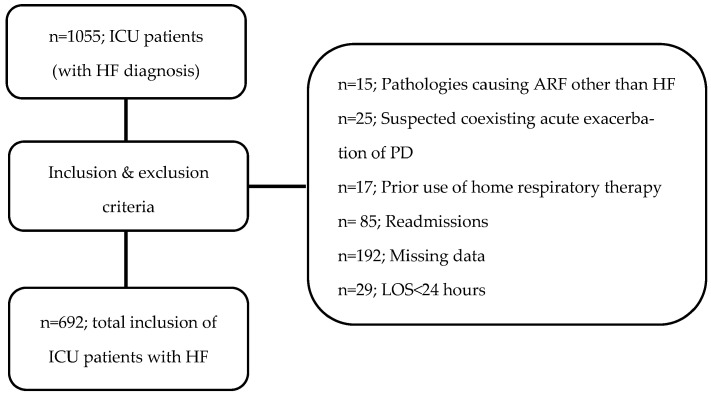
Flow diagram of the study. ICU: intensive care unit, ARF: acute respiratory failure, PD: pulmonary disease, LOS: length of stay.

During the specified period, a total of 1055 patients were admitted to the ICU due to HF. After applying the exclusion criteria and accounting for missing data, the final study cohort comprised 692 patients (Figure 1). The distributions of patients across the groups were as follows: T-NR (*n* = 280, 40.5%), T-O2 (*n* = 220, 31.8%), T-NIMV (*n* = 99, 14.3%), and T-IMV (*n* = 93, 13.4%).

The mean age of the patients was 69.15 ± 15.9 years, and 434 patients (62.7%) were male. The most common comorbidity was HT (68.6%), followed by DM (44.2%), CKD (39.3%), PD (22.5%), CVD (12.6%), and malignancy (9.2%). An ischemic etiology was identified in 297 patients (42.9%), while 395 patients (57.1%) were admitted with a non-ischemic etiology. The mean APACHE II score was 16.08 ± 6.09 and the mean uCCI score was 3.53 ±1.35. Regarding interventions, vasopressor/inotropic therapy was administered to 254 patients (36.7%). Additionally, 150 patients (21.7%) had an ICD, 38 patients (5.5%) had CRT, 2 patients (0.3%) received IABP, and 2 patients (0.3%) received ECMO. Notably, all patients who received ECMO or IABP were in the T-IMV group.

In the inter-group comparisons, the prevalence of CKD was higher in the T-IMV group, while patients in the T-NIMV group had higher rates of PD (*p* < 0.01). Patients in the T-IMV group also exhibited higher APACHE II and uCCI scores (*p* = 0.01), as well as higher rates of vasopressor/inotropic therapy and cardiac interventions (*p* < 0.05). Furthermore, EF was significantly lower in the T-IMV group than in the other groups (*p* = 0.05), whereas PAP was significantly higher in the T-NIMV group (*p* = 0.01). Finally, the T-IMV group had significantly longer ICU and hospital LOS and higher mortality rates the other groups (*p* < 0.05) (Table 1).

In the inter-group analysis of laboratory parameters, the T-IMV group had significantly higher levels of NT-proBNP, LAC, Cre, and TBIL and lower levels of Alb than the other groups at both admission—T0 and discharge—T1 (*p* < 0.05) (Table 2).

A comparison of survivors and non-survivors revealed that the prevalence of CKD, APACHE II and uCCI scores, as well as the rate of vasopressor/inotropic therapy, were significantly higher in the non-survivor group (*p* < 0.05). Furthermore, non-survivors had prolonged hospital and ICU stays, and their PAP and EF values were significantly lower (*p* < 0.05) (Table 3).

Compared to survivors, non-survivors exhibited significantly higher levels of NT-proBNP, LAC, Cre, and TBIL and lower levels of Alb at both T0 and T1 (*p* < 0.05) (Table 4).

Regression analysis was performed to identify factors that were independently associated with mortality. Parameters that showed significance in the comparisons by survival status were first included in the univariable regression analysis, followed by multivariable analysis. In univariable logistic regression analyses, the APACHE II score, the use of vasopressor/inotropic therapy, LAC-T0, cre-T0, Alb-T0 and logarithmically transformed (ln)NT-proBNP-T0 levels were significantly associated with ICU mortality. EF showed a borderline association with mortality in the univariable model (*p* = 0.05). However, in the multivariable model, only the APACHE II scores, the use of vasopressor/inotropic therapy, ln(NT-proBNP) and LAC-T0 levels remained independently associated with mortality. (Table 5). All variables demonstrated VIF values below accepted thresholds, indicating no clinically relevant multicollinearity. Conversely, uCCI, PAP, and TBIL were not associated with mortality in either univariable or multivariable regression analyses (*p* > 0.05).

## 4. Discussion

Our study demonstrated that patients in the IMV group had higher rates of kidney disease, as well as significantly lower EF and PAP values. These patients also required vasopressor/inotropic support more frequently, had longer hospital stays and exhibited higher mortality rates, higher APACHE II scores and higher uCCI values. Laboratory analyses showed that NT-proBNP, LAC, Cre, and TBIL levels were consistently higher in the IMV group at both admission and discharge, while Alb levels were lower. Multivarible analysis revealed that APACHE II scores, vasopressor/inotropic use, and LAC and NT-proBNP) levels were independently associated with mortality.

In critically ill patients with HF, the choice of respiratory treatment methods, ranging from oxygen therapy to non-invasive or invasive MV, is extremely important [2,4,7,12]. However, this decision-making process is often complex due to the clinical severity and hemodynamic fragility of these patients. NIMV in HF has been shown to improve oxygenation, plays an effective role in correcting hypercapnia, and alleviates respiratory distress [4,5,13,14]. Furthermore, NIMV has been shown to reduce the risk of intubation, particularly in high-risk populations [1,5]. IMV may be necessary in cases of more severe respiratory failure. Despite this, IMV in HF patients is known to be associated with high mortality rates and prolonged lengths of stay [1]. Additionally, however, the positive pressure applied during MV in HF patients can lead to significant changes in intrathoracic pressure and subsequent hemodynamic instability in this patient group [13]. Our results demonstrated a clear mortality gradient, whereby patients undergoing IMV had higher mortality rates and longer ICU and hospital LOS. Importantly, the observed differences in mortality and other clinical outcomes across respiratory support modalities should not be interpreted as evidence of a causal effect of the modality itself. In our study, the choice of respiratory support was driven by clinical judgment and underlying disease severity, resulting in substantial confounding by indication. The marked mortality observed in the IMV group, compared with the absence of mortality in the T-NR group, highlights that IMV reflects severe clinical and hemodynamic compromise. Patients in the T-NR group were primarily managed for hemodynamic instability rather than respiratory failure, whereas IMV was generally initiated in the context of severe clinical illness. Patients requiring more advanced respiratory support, particularly IMV, represented a more critically ill population with a higher baseline risk of poor outcomes. These findings should be interpreted in the context of confounding by indication, as the higher mortality and prolonged LOS observed in the IMV group are likely driven by greater clinical severity and disease burden rather than the advanced respiratory therapy itself. This interpretation is supported by the T-IMV group having significantly higher APACHE II scores and uCCI, as well as exclusively using invasive interventions such as ECMO or IABP and having an increased need for vasopressor/inotropic support. These findings suggest that patients were not only more severely ill but also experienced catastrophic decompensation that precluded oxygen therapy or NIMV use. Furthermore, reflecting the link between severe hemodynamic dysfunction and poor clinical outcomes, we found that the use of vasopressor/inotropic therapy was associated with mortality, independently of the type of respiratory support administered.

A high uCCI indicates significant comorbidity and reduced physiological reserve across multiple organ systems [15], while the APACHE II score is widely accepted as the primary indicator of overall physiological impairment and disease severity in ICU [16]. Studies have shown that the APACHE II score successfully predicts short-term mortality in ICU patients with HF [11,16,17]. In line with previous research, our study found that uCCI and APACHE II scores were significantly higher in the non-survivor group across the entire cohort. Most importantly, multivariable regression analysis revealed that the APACHE II score was independently associated with mortality. We believe that a high APACHE II score provides an effective, dynamic snapshot of acute physiological injury and accompanying organ failure. Although the APACHE II score was not primarily designed for HF assessment, it may indicate that the systemic consequences of HF in critically ill patients are a more important cause of death than the primary cardiac pathology itself. Furthermore, the high uCCI scores observed in our non-survivors confirm the association between multimorbidity and poor prognosis in this population [18]. In cases of acute decompensated HF with high uCCI, already damaged organs may deteriorate further into failure, leading to an increased need for IMV and higher mortality rates. However, our multivariable regression analysis did not identify a significant independent relationship between uCCI and mortality. We hypothesized that this may be because uCCI measures overall disease burden but does not necessarily reflect the current severity or stability of underlying conditions.

Age, sex, and the presence of comorbidities are recognized as key factors influencing the prognosis of HF [8,19,20,21]. Advanced age increases the risk of HF, particularly HF with preserved EF (HFpEF), by causing increased afterload due to arterial stiffness [22], decreased diastolic function [23], reduced beta-adrenergic sensitivity [24], and pro-inflammation associated with cellular senescence [25]. Sex differences also play a significant role in the HF phenotype; while women are more likely to develop HFpEF, they tend to have lower mortality rates than men in the context of HF with reduced EF (HFrEF) at similar EF [20,21]. In elderly patients especially, comorbidities such as HT, DM, CKD, atrial fibrillation, stroke, malignancies, and chronic obstructive pulmonary disease (COPD) are known to contribute directly to the development or complication of HF [8,19,26]. HT is a direct cause of HF, while impaired renal function leads to fluid overload and deteriorating cardiac function, DM contributes via cardiomyopathy and diastolic dysfunction, and the presence of COPD complicates both diagnosis and treatment, thereby increasing mortality [8,27,28,29]. Additionally, therapies used in HF management, such as angiotensin receptor antagonists and diuretics, may adversely affect renal function [28,30]. In our study, we found no significant association between age or sex and either respiratory support modalities or mortality. We believe that the severity of HF in our cohort may be more critical to mortality than age or sex. This suggests that, when a patient is critically ill enough to require ICU management, biological factors may be less relevant to the clinical outcome compared to hemodynamic stability and organ perfusion.

We found higher PD rates in patients treated with NIMV. This may be due to the tendency to prioritize NIMV treatment in patients with underlying PD, particularly in cases of hypercapnia. Although we excluded patients with acute restrictive or obstructive PD from the study, clinicians may still prefer NIMV as the initial respiratory support for HF patients with baseline pulmonary comorbidities. While CKD was excluded from the regression model for the methodological reasons described above, a higher prevalence of CKD was observed in the IMV group and in non-survivors. This finding is consistent with previous research and suggests an important interaction between the heart and kidneys. It indicates that a severe HF is often associated with renal dysfunction, or conversely, that renal dysfunction may exacerbate HF [8,19,28]. The higher prevalence of CKD in our IMV group may be attributed to volume overload which worsens pulmonary edema and metabolic acidosis which impairs respiratory muscle function and necessitates invasive support.

In our study, patients undergoing IMV were found to be in the most critical clinical state of the respiratory treatment groups, yet their PAP values were lower than those in the NIMV group. While this finding may appear to contradict the expectation of high PAP in IMV patients, it is a key clinical finding that can be explained by HF stages and hemodynamic principles. In advanced left HF, reduced ventricular pump function leads to a decrease in stroke volume. This condition can create a ‘pseudo-normalization’ of measured pulmonary pressures due to biventricular failure, even if pulmonary vascular resistance remains high [31]. In their study on right ventricular (RV) function in HF, Guazzi et al. also suggested that, in the end-stages of pulmonary hypertension, the RV can no longer overcome the afterload and loses its contractile strength [32]. Therefore, the lower PAP measured in our IMV group likely reflects very low cardiac output or significant RV failure. This underscores a vital message for ICU practitioners: echocardiographic PAP measurements must be interpreted with caution, as lower values may paradoxically signal end-stage cardiac collapse and systemic failure rather than hemodynamic stability. Furthermore, the accuracy of the PAP measurements obtained using echocardiographic methods, may have been affected by image quality and technical factors. It has been reported that HF of ischemic etiology may have a more aggressive prognosis and higher mortality than non-ischemic HF, due to morbidities arising from recurrent ischemic events or the development of ventricular arrhythmias [33,34]. In unstable patients with ischemic HF, the requirement for IMV may be greater due to acute cardiogenic shock and pulmonary edema [34,35]. However, in our study, we found no significant association between HF etiology and either respiratory treatment modalities or mortality. We suggest that HF etiology may indirectly influence mortality or respiratory support through the severity of the clinical presentation and concomitant comorbidities, rather than directly. For example, a patient with fulminant shock induced by an ischemic event may require IMV, whereas a patient with slowly decompensating HF due to underlying hypertension, arrhythmia or pulmonary issues may be a more suitable candidate for NIMV. Furthermore, although it has been reported that patients with reduced EF carry a higher risk of death [17,36], we found lower EF values in the IMV and non-survivor groups; however, this association was not significant in the multivariable analysis. We believe that the prognosis of patients in the ICU is more closely related to the degree of systemic collapse than to the specific HF phenotype or the etiology of the cardiomyopathy.

NT-proBNP is recognized as a crucial biomarker for assessing myocardial wall stress and is widely utilized as a prognostic indicator in HF [3,9]. Elevated LAC levels reflect impaired tissue perfusion and shock states, while increased in Cre levels indicate changes in renal perfusion. Additionally, elevated TBIL levels may indicate liver dysfunction caused by congestion [10,30,37]. Albumin, a negative acute-phase reactant, exhibits decreased levels in cases of reduced liver synthesis, severe inflammation and a systemic catabolic response [38]. In our study, patients receiving IMV had higher levels of NT-proBNP, LAC, Cre and TBIL and lower levels of Alb at both admission and discharge, compared to patients receiving non-invasive respiratory therapy. These findings clearly demonstrate cardiac, renal and hepatic interactions, which reflect the critical illness status of HF patients requiring IMV. Most importantly, multivariable regression analysis revealed that both LAC and NT-proBNP values at admission were independently associated with mortality. These markers reflect early metabolic and hemodynamic instability in our severely ill HF patients that signals impending clinical deterioration. The observation that NT-proBNP and LAC levels were even higher at discharge or immediately prior to death in the IMV group suggests severe hemodynamic deterioration and shock toward the time of the outcome. To avoid outcome leakage, discharge values were not included in the regression model and are therefore interpreted descriptively. In our cohort, higher LAC levels at ICU admission were predominantly observed in patients requiring IMV, who also exhibited more severe hemodynamic compromise. This finding suggests that LAC elevation in our critically ill HF patients reflects global hypoperfusion. The independent association between LAC and mortality may support its role as a marker of severe systemic hypoxia, metabolic stress and adverse outcome in this population. Although lower Alb levels were significantly associated with mortality in univariable analyses, this association did not remain statistically significant after multivariable analysis. Hypoalbuminemia in HF patients may reflect advanced disease, congestion-related hepatic dysfunction, and a heightened inflammatory state, all of which are linked to adverse outcomes. Furthermore, a decrease in albumin levels may contribute to clinical deterioration by reducing oncotic pressure and exacerbating peripheral and pulmonary oedema.

Our study has several strengths. First, it has a large sample size. Secondly, a ‘cleaner’ cohort was created by excluding patients with re-admissions, those receiving long-term oxygen or MV therapy, and those with pathologies other than HF that could cause acute respiratory failure. These exclusions enabled us to make detailed categorical comparisons of different respiratory treatment methods and perform robust multivariable regression analyses. However, our study has some limitations. Due to its retrospective nature, there is a risk of data loss and selection bias. Furthermore, as it is a single-center study, this limits the generalizability of our findings. Although the retrospective design inherently carries a risk of missing data, we minimized the impact of missingness by applying strict inclusion criteria that required complete records for all primary variables. However, we acknowledge that this approach may introduce a degree of selection bias, as patients with the most critical or unstable conditions may have had incomplete imaging or laboratory data due to technical or logistical constraints in the ICU setting. Since interventions such as ECMO and IABP were only applied to the IMV group, it was not possible to assess their independent effects on the outcome. Additionally, PAP was measured using echocardiography instead of right heart catheterization. Although echocardiography is a widely used, non-invasive, and valuable tool for assessing PAP, the accuracy of its measurements may be affected by various technical factors. Finally, due to the retrospective and non-randomized design, specific indications for respiratory treatment modalities could not be uniformly categorized and were based on clinical decision-making. The absence of a standardized protocol for respiratory therapies may have introduced variability into our results and contributed to indication bias.

## 5. Conclusions

In conclusion, this study demonstrates that HF patients requiring IMV are faced with a critical clinical course characterized by severe respiratory failure, significant hemodynamic abnormalities, and multiple organ dysfunction, all of which are associated with poor outcomes. The requirement for IMV reflects extreme clinical severity and hemodynamic instability, and should be interpreted as a marker of advanced disease rather than a driver of mortality. Furthermore, an unfavorable biochemical profile manifested by persistent elevation of NT-proBNP, LAC, Cre, and TBIL levels, alongside hypoalbuminemia, at both admission and discharge supports these poor clinical outcomes, as reflecting extensive cardiac, renal, and hepatic involvement. Multivariable analysis demonstrated that mortality was independently associated with overall disease severity, as reflected by the APACHE II score. This indicates that critically ill HF patients should not be evaluated solely based on cardiac criteria. Patient management should therefore be based on a comprehensive assessment that includes disease severity scores, organ perfusion markers and inflammatory status. A multidisciplinary approach should also be adopted to support the heart and the systemic organs that it can no longer perfuse adequately.

## Figures and Tables

**Table 1:** Comparison of patient characteristics according to respiratory treatment modalities.

		T-NR Group *n* = 280	T-O_2_ Group *n* = 220	T-NIMV Group *n* = 99	T-IMV Group *n* = 93	*p*
Age (years)		66.02 ± 15.87	68.45 ± 14.89	70.90 ± 14.29	68.21 ± 14.90	0.607
Sex	Male	177 (63.2%)	144 (65.5%)	47 (47.5%)	61 (63.7%)	0.141
	Female	103 (36.8%)	76 (34.5%)	52 (52.5%)	32 (34.3%)	
Comorbidities	HT	186 (66.4%)	152 (69.1%)	73 (73.7%)	64 (68.8%)	0.324
	DM	123 (43.9%)	89 (40.5%)	51 (51.5%)	43 (46.2%)	0.214
	CKD	75 (26.8%)	93 (42.3%)	49 (49.5%)	55 (59.1%)	<0.01
	PD	59 (21.9%)	75 (34.1%)	44 (44.4%)	26 (28%)	<0.01
	CVD	29 (10.4%)	30 (13.6%)	15 (15.2%)	13 (14%)	0.524
	Malignancy	16 (5.7%)	26 (11.8%)	9 (9.1%)	13 (14%)	0.394
HF-Etiology	Ischemic	128 (43.7%)	92 (41.8%)	36 (36.7%)	41 (44.1%)	0.12
	Non-ischemic	152 (54.3%)	128 (59.2%)	63 (63.3%)	52 (55.9%)	
APACHE II Score		12.53 ± 4.08	15.13 ± 5.25	18.96 ± 6.71	25.92 ± 6.86	0.01
uCCI		3.03 ± 1.06	3.76 ± 1.42	3.94 ± 1.32	4.08 ± 1.49	0.01
PAP mmHg		40.59 ± 13.41	43.34 ± 14.24	49.15 ± 15.40	48.16 ± 14.07	0.01
EF%		31.21 ± 13.16	31.39 ± 12.98	31.59 ± 14.22	27.81 ± 13.50	0.050
Cardiac Interventions	ICD	58 (20.5%)	49 (22.7%)	18 (18.8%)	25 (26.8%)	0.066
	CRT	14 (5%)	13 (6%)	3 (3.1%)	8 (8.7%)	0.426
	IABP	0	0	0	2 (2.2%)	-
	ECMO	0	0	0	2 (2.2%)	-
Vasopressor/inotropic therapy		40 (14.28%)	77 (35%)	51 (51.51%)	85 (91.39%)	<0.01
LOS-ICU (days)		7.12 ± 6.84	9.48 ± 7.91	11.62 ± 9.34	15.46 ± 19.62	<0.01
LOS-H (days)		12.59 ± 10.01	15.88 ± 10.81	19.69 ± 14.40	20.90 ± 20.89	<0.01
Outcome	Death	0	2 (0.9%)	15 (15.6%)	84 (90.3%)	<0.01
	Discharge	280 (100%)	218 (99.1%)	84 (84.4%)	9 (9.7%)	

T-NR: treatment—non respiratory, T-O_2_: treatment—oxygen, T-NIMV: treatment—non-invasive mechanical ventilation, T-IMV: treatment—invasive mechanical ventilation, HT: hypertension, DM: diabetes mellitus, CKD: chronic kidney disease, PD: pulmonary disease, CVD: cerebrovascular disease, HF: heart failure, APACHE II: Acute Physiology and Chronic Health Evaluation, uCCI: updated Charlson Comorbidity Index, PAP: pulmonary artery pressure, EF: ejection fraction, ICD: implantable cardioverter defibrillator, ECMO: extracorporeal membrane oxygenation, CRT: Cardiac resynchronization therapy, IABP: intra-aortic balloon pump, LOS: length of stay, ICU: intensive care unit, H: hospital. Continuous variables are presented as mean ± standard deviation, categorical variables as n (%).

**Table 2:** Comparison of patients’ laboratory values according to respiratory treatment modalities.

	T-NR Group *n* = 280	T-O_2_ Group *n* = 220	T-NIMV Group *n* = 99	T-IMV Group *n* = 93	*p*
NT-proBNP-T0 (pg/mL)	5115 (2076.50–10,499.50)	7956 (3900–17,045.70)	11,000 (4940–26,710)	11,027 (5751–23,909)	<0.001
NT-proBNP-T1 (pg/mL)	3200 (1205–7481)	4827 (2015.50–10,490)	8850 (3253.50–17,485)	23,578 (11,799.50–35,000)	<0.001
LAC-T0 (mmol/L)	1.80 (1.30–2.50)	1.81 (1.31–2.50)	1.70 (1.35–2.41)	2.04 (1.60–3.16)	0.006
LAC-T1 (mmol/L)	1.40 (1.04–1.95)	1.40 (1.04–1.80)	1.30 (1.10–1.71)	5.37 (2.50–10.56)	<0.001
Cre-T0 (mg/dL)	1.00 (0.83–1.40)	1.20 (0.90–1.60)	1.40 (1.00–1.89)	1.53 (1.11–2.00)	<0.001
Cre-T1 (mg/dL)	1.00 (0.86–1.32)	1.18 (0.90–1.50)	1.50 (1.00–1.90)	2.00 (1.39–2.66)	<0.001
Alb-T0 (g/L)	39.00 (35.00–42.00)	37.00 (34.00–41.00)	36.00 (34.00–39.00)	34.00 (31.00–39.00)	<0.001
Alb-T1 (g/L)	39.00 (35.00–41.00)	36.00 (33.00–39.00)	34.00 (31.00–38.00)	28.00 (24.00–33.00)	<0.001
TBIL-T0 (mg/dL)	0.80 (0.60–1.27)	0.80 (0.51–1.40)	0.90 (0.60–1.65)	1.08 (0.80–1.80)	0.001
TBIL-T1(mg/dL)	0.69 (0.41–1.15)	0.70 (0.40–1.00)	1.00 (0.60–1.45)	1.79 (0.96–2.82)	<0.001

T-NR: treatment—non respiratory, T-O_2_: treatment—oxygen, T-NIMV: treatment—non-invasive mechanical ventilation, T-IMV: treatment—invasive mechanical ventilation, T0: at admission; T1: at ICU discharge or the last measurement before death, NT-proBNP: N-terminal pro-B-type natriuretic peptide, LAC: lactate, Cre: creatinine, Alb: albumin, TBIL: total bilirubin. Continuous variables are presented as median [IQR].

**Table 3:** Comparison of patient characteristics based on survival status.

		Survivor *n* = 591	Non-Survivor *n* = 101	*p*
Age (year)		67.10 ± 15.29	69.01 ± 15.54	0.45
Sex	Male	366 (84.3%)	68 (15.7%)	0.33
	Female	225 (87.2%)	33 (12.8%)	
Comorbidities	HT	408 (85.9%)	67 (14.1%)	0.58
	DM	260 (85%)	46 (15%)	0.772
	CKD	211 (77.6%)	61 (22.4%)	<0.001
	PD	178 (87.3%)	26 (22.7%)	0.373
	CVD	72 (82.8%)	15 (17.2%)	0.455
HF: Etiology	Malignancy	50 (78.1%)	14 (21.9%)	0.079
	Ischemic	254 (85.5%)	43 (14.5%)	0.94
	Non-ischemic	337 (85.3%)	58 (14.7%)	
APACHE II Score		14.24 ± 5.01	26.84 ± 6.64	0.02
uCCI		3.42 ± 1.27	4.20 ± 1.57	0.01
PAP (mmHg)		48.04 ± 13.55	42.94 ± 14.47	0.01
EF%		31.48 ± 13.20	27.28 ± 13.61	0.04
Cardiac Interventions	ICD	126 (84%)	24 (16%)	
	CRT	27 (71.1%)	11 (28.9%)	0.582
	IABP	0	2	
	ECMO	0	2	
Vasopressor/inotropic therapy		157 (61.8%)	97 (38.2%)	<0.001
LOS-ICU (days)		9.02 ± 7.61	15.46 ± 19.62	<0.001
LOS-H (days)		14.95 ± 11.05	20.05 ± 21.42	<0.001

HT: hypertension, DM: diabetes mellitus, CKD: Chronic kidney disease, PD: pulmonary disease, CVD: cerebrovascular disease, HF: heart failure, APACHE II: Acute Physiology and Chronic Health Evaluation, uCCI: updated Charlson Comorbidity Index, PAP: pulmonary artery pressure, EF: ejection fraction, ICD: implantable cardioverter defibrillator, ECMO: extracorporeal membrane oxygenation, CRT: Cardiac resynchronization therapy, IABP: intra-aortic balloon pump, LOS: length of stay, ICU: intensive care unit, H: hospital. Continuous variables are presented as mean ± standard deviation, categorical variables as n (%).

**Table 4:** Comparison of laboratory parameters based on survival status.

	Survivor *n* = 591	Non-Survivor *n* = 101	*p*
NT-proBNP-T0 (pg/mL)	7008 (2935–13,504)	12,010 (5780–24,282)	<0.001
NT-proBNP-T1 (pg/mL)	4306 (1566–9500)	25,000 (13,662–35,000)	<0.001
LAC-T0 (mmol/L)	1.80 (1.30–2.50)	2.04 (1.70–3.44)	<0.001
LAC-T1(mmol/L)	1.40 (1.00–1.80)	6.00 (3.00–11.00)	<0.001
Cre-T0 (mg/dL)	1.13 (0.89–1.52)	2.00 (1.48–2.76)	<0.001
Cre-T1 (mg/dL)	1.10 (0.89–1.50)	2.04 (1.70–3.44)	<0.001
Alb-T0 (g/L)	38.00 (35.00–41.00)	34.00(30.00–39.00)	<0.001
Alb-T1 (g/L)	37.00 (33.00–40.00)	28.00 (23.00–34.00)	<0.001
TBIL-T0 (mg/dL)	0.80 (0.55–1.30)	1.10 (0.80–2.00)	<0.001
TBIL-T1 (mg/dL)	0.70 (0.44–1.10)	1.82 (1.00–2.92)	<0.001

T0: at admission; T1: at ICU discharge or the last measurement before death, NT-proBNP: N-terminal pro-B-type natriuretic peptide, LAC: lactate, Cre: creatinine, Alb: albumin, TBIL: total bilirubin. Continuous variables are presented as median [IQR].

**Table 5:** Logistic regression analysis of parameters associated with mortality.

	Univariable			Multivariable		
	Odds Ratio	95% CI	*p*	Odds Ratio	95% CI	*p*
APACHE II Score	1.36	1.95–1.442	<0.001	1.35	1.26–1.45	<0.001
Vasopressor/inotrope therapy	67.03	24.57–185.25	<0.001	49.51	14.12–173.64	<0.001
uCCİ	1.41	0.91–1.64	0.162	-	-	-
PAP mmHg	0.96	0.83–0.99	0.06	-	-	-
EF%	0.98	0.96–0.99	0.05	-	-	-
Alb-T0 (g/L)	0.89	0.85–0.93	<0.001	0.98	0.92–1.05	0.641
ln(NT-proBNP)-T0 (pg/mL)	1.71	1.37–2.14	<0.001	1.60	1.09–2.36	0.017
Cre-T0 (mg/dL)	1.38	1.11–1.72	0.004	0.90	0.58–1.40	0.635
LAC-T0 (mmol/L)	1.42	1.25–1.62	<0.001	1.33	1.04–1.70	0.021
TBIL-T0 (mg/dL)	1.29	0.78–1.50	0.200	-	-	-

APACHE II: Acute Physiology and Chronic Health Evaluation, T0: admission time, uCCI: updated Charlson Comorbidity Index, PAP: pulmonary artery pressure, EF: ejection fraction, ln(NT-proBNP): logarithmically transformed N-terminal pro-B-type natriuretic peptide, Cre: creatinine, Alb: albumin, LAC: lactate, TBIL: total bilirubin, AUC: 0.971, Nagelkerke R^2^: 0.739, Hosmer–Lemeshow test: χ^2^ = 5.12, df = 8, *p* = 0.744.

## Data Availability

The data underlying this article will be shared upon reasonable request to the corresponding author.

## Abbreviations

The following abbreviations are used in this manuscript:

HF	Heart Failure
ICU	Intensive Care Unit
IMV	Invasive Mechanical Ventilation
NIMV	Non-Invasive Mechanical Ventilation
APACHE II	Acute Physiology and Chronic Health Evaluation II
NT-proBNP	N-terminal pro-B-type Natriuretic Peptide
O_2_	Oxygen
uCCI	Updated Charlson Comorbidity Index
LOS	Length of Stay
HT	Hypertension
DM	Diabetes Mellitus
CKD	Chronic Kidney Disease
PD	Pulmonary Disease
CVD	Cerebrovascular Disease
PAP	Pulmonary Artery Pressure
EF	Ejection Fraction
IABP	Intra-Aortic Balloon Pump
ICD	Implantable Cardioverter-Defibrillator
ECMO	Extracorporeal Membrane Oxygenation
CRT	Cardiac Resynchronization Therapy
LAC	Lactate
Cre	Creatinine
Alb	Albumin
TBIL	Total Bilirubin
